# A Description of the Bones, Together with Their Several Connexions with Each Other and with the Muscles; Especially Adapted for Students in Anatomy

**Published:** 1837-04

**Authors:** 


Art. XIV.—*A Description of the Bones, together with their several Connexions with each other and with the Muscles; specially adapted for Students in Anatomy.* By John F. South, Assistant Surgeon to St. Thomas's Hospital. Third Edition.—*London*, 1837. pp. 146.

We have examined this third edition of Mr. South's "Description of the Bones" with a great deal of pleasure, and strongly recommend it to students as a much improved work. The great improvements are the introduction of minute, but accurate and distinct wood-engravings of the several bones by Branston, exhibiting the different foramina, angles, tubercles, &c. accurately distinguished, and numbered, with reference to the letter-press, after the manner of Paxton's Anatomy, but much more distinctly and accurately. As the bones necessarily form the very commencement of the study of anatomy, and without a correct knowledge of which, of their processes, foramina, tubercles, elevations, depressions, angles, and surfaces, it is impossible to make any permanent advancement in a knowledge of the other parts of the body, we have always felt persuaded that it is such a work as this that ought first to be placed in the hands of the student; not with the expectation that it, even when closely studied, would render him master of the *whole* of the anatomy of the parts, but with the confidence that it would be one of the best means of engaging his attention to those parts, which are proverbially considered dry and uninteresting, however important may be a correct knowledge of them. Who, for instance, when he first took up the sphenoid bone, or the petrous portion of the temporal, or the ethmoid, was ever able to recognize their several parts, by mere printed descriptions, without great labour, and perhaps again and again throwing them aside in disgust, and almost despair? It is in remedying this difficulty, and preventing the loss of time and labour attendant upon it, that Mr. South's work will be found useful; and we therefore recommend it strongly to the attention of pupils and the masters of pupils.

